# Interfacial jamming reinforced Pickering emulgel for arbitrary architected nanocomposite with connected nanomaterial matrix

**DOI:** 10.1038/s41467-020-20299-6

**Published:** 2021-01-04

**Authors:** Yuanyuan Zhang, Guangming Zhu, Biqin Dong, Feng Wang, Jiaoning Tang, Florian J. Stadler, Guanghui Yang, Shuxian Hong, Feng Xing

**Affiliations:** 1 Department of Civil and Transportation Engineering, Guangdong Province Key Laboratory of Durability for Marine Civil Engineering, 518060 Shenzhen, People’s Republic of China; 2 College of Materials Science and Engineering, Shenzhen University, 518071 Shenzhen, People’s Republic of China; 3 Department of Materials Science and Engineering, City University of Hong Kong, 83 Tat Chee Avenue, Hong Kong SAR, People’s Republic of China

**Keywords:** Optical properties and devices, Two-dimensional materials, Self-assembly

## Abstract

Three-dimensional (3D) nanocomposite (NC) printing has emerged as a major approach to translate nanomaterial physical properties to 3D geometries. However, 3D printing of conventional NCs with polymer matrix lacks control over nanomaterial connection that facilitates maximizing nanomaterial advantages. Thus, a printable NC that features nanomaterials matrix necessitates development, nevertheless, faces a challenge in preparation because of the trade-off between viscosity and interfacial stability. Here, we develop viscoelastic Pickering emulgels as NC inks through jamming nanomaterials on interfaces and in continuous phase. Emulgel composed of multiphases allow a vast range of composition options and superior printability. The excellent attributes initiate NC with spatial control over geometrics and functions through 3D printing of graphene oxide/phase-change materials emulgel, for instance. This versatile approach provides the means for architecting NCs with nanomaterial continuous phase whose performance does not constrain the vast array of available nanomaterials and allows for arbitrary hybridization and patterns.

## Abstract (web summary)

Nanocomposite (NC) printing emerged as a major approach to translate nanomaterial properties to 3D geometries but printing of conventional NCs lacks control over nanomaterial connection. Here, the authors develop viscoelastic Pickering emulgels as NC inks through jamming nanomaterials on interfaces and in continuous phase

## Introduction

**Fig. 1:**
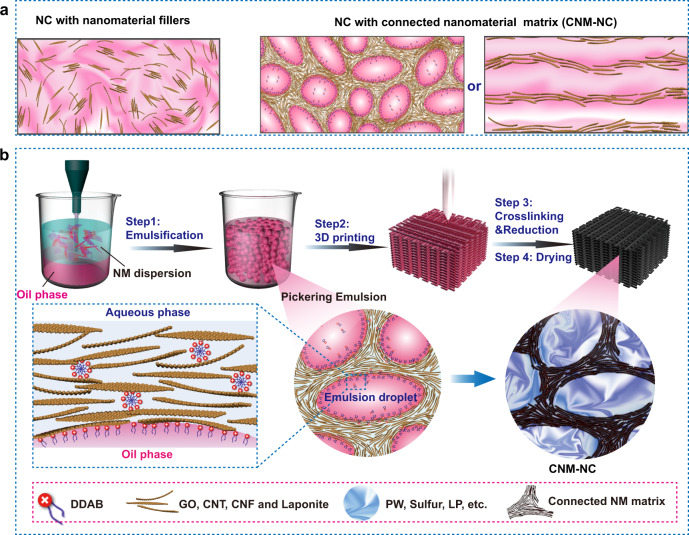
Schematic illustrations of CNM-NC 3D printing. **a** The illustrations depict the traditional nanocomposite (NC) and NC with a connected nanomaterial matrix. **b** The illustration of 3D printing of Pickering emulgel. Step 1: The mixture of aqueous nanomaterial (NM) dispersion and oil were emulsified into Pickering emulgel using a homogenizer. The jamming of droplets and nanomaterials results in suitable rheology for direct ink printing. Step 2: The obtained emulgel is extruded into filaments through a nozzle and printed layer-by-layer into various patterns. Crosslinking and the concurrently occurred reduction at step 3, as well as the following drying in step 4 are conducted to obtained stable nanocomposites. The nanocomposites allow the hybridization of water-soluble and non-water-soluble species from both phases.

Three-dimensional (3D) nanocomposite (NC) printing offers a programmable, facile, and flexible manner to translate the inherent physical properties of nanomaterials into 3D topologies and geometries, which are appreciated by various sectors, such as microelectronics^1^, aerospace^2^, engineered composites^3^, energy storage^4^, sensors^5,6^, and healthcare^7–11^. Conventionally, NCs gain 3D printability by incorporating nanomaterials into a printable matrix, such as hydrogels, polymer, and resin^9,12^. However, such type of NCs lacks tight control over the nanomaterial connection^13^, even though the connection can maximize nanomaterial advantages, such as electrical and thermal conductivity^14,15^. In terms of this requirement, NC that contains a connected nanomaterial matrix (shorted as CNM-NC for convenient expression) emerges as a new type NC (Fig. 1a), which shows the benefits in thermal management^16,17^, thermal energy storage and harvesting^15,18,19^, supercapacitors^14^, and Li–S batteries^20^. However, their manufacturing technologies are limited to post-infiltration and layer-by-layer casting, which hardly engineer them into 3D structures and have hampered their rapid innovations and broad applications^18,19,21^. Therefore, a 3D printing strategy is required to reveal defined geometry and functionality distribution; however, it faces stringent challenges in preparing printable multiphase inks with nanomaterial continuous phase or solidifying printed structures.

Direct ink writing (DIW) is promising for CNM-NC 3D printing because extrusion molding is possible without additional manufacturing steps (e.g., photopolymerization or thermoforming)^22^. The primary challenge for this method is to design viscoelastic inks that possess a shear-thinning behavior to facilitate flow under pressure and reach an appropriate storage modulus to retain the shape after printing^14^. The most widely developed nanomaterial-contained multiphase inks for DIW are emulsion systems^23–25^. While emulsions involving viscous additives and a large number of co-emulsifiers can fulfill those requirements, however, those additives unavoidably prevent nanomaterial connection^23,25,26^. Particle-stabilized Pickering emulsions are the possible candidates for CNM-NC inks. The gelation of Pickering emulsions into printable multiphase emulsion gel (emulgels) without using viscous additives is still a challenge. A possible solution is increasing the disperse phase volume to above 74% to enhance the van der Waals attraction between droplets, thereby improving the viscosity. The produced high-internal-phase emulsion (HIPE) is rudimentarily printable but clearly suboptimal^27–30^. In addition, HIPE is hypercritical to stabilizer surface properties, thus, limiting the broader use. Thus, intensive efforts to developing Pickering emulgel with wide-ranged tailorable composition and concurrently possessing viscoelastic and interfacial stability are required^31^.

Some concentrated nanomaterial dispersions, such as cellulose nanofibers^32^, graphene oxide (GO)^33^, MXene^34^, and complexed nanomaterials^35^, have been exploited for DIW owing to their appreciably high viscoelasticity. Thus, an alternative strategy to develop a desirable ink is using viscous nanomaterial dispersions as Pickering surfactants. However, the absorption of nanomaterials onto oil/water (O/W) interfaces in viscous dispersions need to overcome the intense attraction between nanomaterials, otherwise resulting in a failure of emulsification^36,37^. Counterion-mediated nanomaterial interfacial assembly has been demonstrated to be a practical approach to increase nanomaterial absorption at liquid/liquid interfaces. This assembly results in the interfacial jamming of nanomaterials, which yields a solid interfacial layer^38–41^. The significantly enhanced interfacial stability makes it a viable approach to preparing viscoelastic Pickering emulgel inks.

In this work, we present interfacial jamming reinforced Pickering emulgel system for complex CNM-NC 3D printing and exploit 3D-printed phase-change material (PCM) composites as a proof to demonstrate advanced functions contributed by this strategy. As illustrated in Fig. 1b, the interfacial jamming induced by electrostatic interactions between nanomaterials and opposite charged surfactants creates stable O/W interfaces, which serve as templates to confine the nanomaterial assembly along the O/W interfaces. Nanomaterial jamming in narrow space between droplets lowers the droplet movements and deformability, thereby increasing the viscosity and stiffness of the emulgel, which ensures an excellent printability of the Pickering emulgel. As a proof-of-concept, we demonstrate the versatile complexation and freeform architecture ability of Pickering emulgel. Through CNM-NC 3D printing, composite PCMs are constructed into various 3D geometries, which allows shape-changing, and furthermore, promises the potential for the applications in flexible/3D devices. More significantly, the 3D-printed woodpile composite PCMs increase light-thermal storage/release to *η* ∼ 97.5%, which is 15% better than bulk counterparts. Such a result demonstrates that the composite PCM performance can be improved by optimizing not only the composition but also the printed geometry, which avoids overcritical requirements on material composition and extends compatibility in multi-material integration. The presented 3D printing strategy promises flexible solutions for complex and programmable thermal management, internal thermal management of devices, and multi-material integrated 3D printing for integrated multifunction structures.

## Results

### Interfacial jamming reinforced Pickering emulgel

The Pickering emulgel composes of viscous continuous nanomaterial phase and dispersion oil phase that contains oppositely charged surfactants. A high-concentration aqueous nanomaterials dispersion of Cellulose nanofibers, nano clay (e.g., laponite), or GO was used as the Pickering surfactants to demonstrate the feasibility and generality of this approach. Didodecyldimethylammonium bromide (DDAB, CAS-Number 3282-73-3), as an example of surfactant, was introduced into the oil phase to favor nanomaterials attraction at the O/W interface. In the presence of DDAB in the oil phase, stable Pickering emulgels were achieved for all compositions tested, but failed when it was absent (Supplementary Figs. 1 and 2), demonstrating the effectiveness of the interfacial assembly approach to prepare Pickering emulgel from viscous nanomaterial dispersions. As a proof-of-concept, GO-based emulgel was systematically investigated to demonstrate the versatility of this approach in the following discussion.

**Fig. 2:**
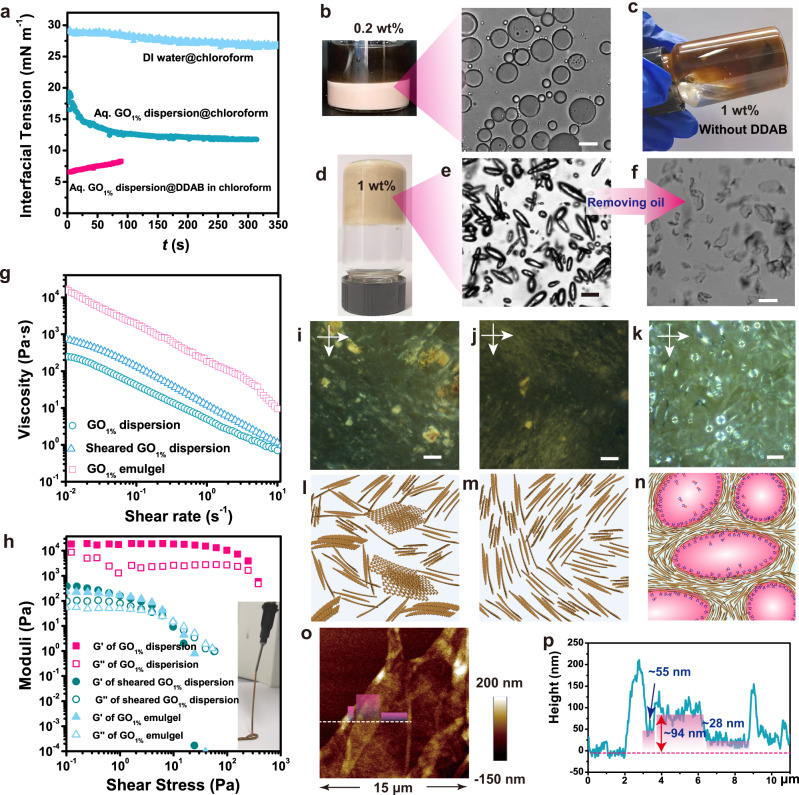
Formation of interfacial jamming stabilized GO/DDAB emulgel inks. **a** Time evolution of interfacial tension of different oil-water systems, including deionized (DI) water@chloroform, aqueous (aq.) GO_1.0%_ dispersion@chloroform, or aq. GO_1.0%_ dispersion@chloroform DDAB solution. **b** Optical and optical microscope (OM) images of GO_0.2%_/Idobenzene (IB) emulsion. **c** Image of IB and aq. GO_1.0%_ solution mixtures that fail in emulsification. **d** Image of GO_1.0%_/DDAB-IB emulsion. **e**, **f** OM images of GO_1.0%_/DDAB-IB emulsion droplets before (**e**) and after removing the oil phase (**f**). Continuous membranes outside droplets are displayed. **g**, **h** Apparent viscosity as a function of shear rate (**g**) and shear storage modulus as a function of shear stress for GO_1.0%_ dispersion and GO_1.0%_/DDAB-IB. *G*’ and *G*” represent the storage modulus and loss modulus, respectively. Inset in (**h**) is a photograph of sustainable GO_1.0%_/DDAB-IB filaments after extrusion. **i**–**n** Polarized optical microscopy (POM) images of GO texture (**i**–**k**) and schematic illustration of GO packing (**l**–**n**) in GO_1.0%_ dispersion with (I, **l**)/without shearing (**j**, **m**) and GO_1.0%_/DDAB-IB emulgel (**k**, **n**). **o** Atomic force microscope (AFM) image of the interfacial membrane that formed at the interface of chloroform DDAB solution (0.1 wt%) and aq. GO solution (0.05 wt%) and **p** the height profile. The heights of 28 nm, 55 nm, 94 nm correspond to one, two, and three folded layers of membrane. Scale bars, 20 μm (**b**, **e**, **f**, **k**), 100 μm (**I**, **j**).

The interfacial stability of GO at the O/W interface in the presence/absence of DDAB was initially examined by pendent drop tensiometry. The interfacial tension (*γ*) between the aqueous GO dispersion and oil phase (without surfactants) was as low as 11.8 mN m^−1^, indicating the remarkable amphiphilicity of GO (Fig. 2a). Accordingly, emulsification was achieved when using GO as the Pickering surfactant at a concentration as low as 0.2 wt% (Fig. 2b), but could not succeed in 3D printing owing to the lack of dimensional stability. Increasing the GO concentration to 1 wt% led to macroscopic phase separation and, thus, to emulsification failure (Fig. 2c). Both results confirm that the GO sheets are confined in the aqueous phase owing to the enhanced interaction and jamming in the highly concentrated GO dispersion rather than locating on the O/W interfaces.

In the presence of 0.1 wt % DDAB, the interfacial tension was reduced to 6.7 mN m^−1^, leading to an improved O/W interfacial stability (Fig. 2a). A stable emulsion was achieved when the GO concentration exceeded 1 wt% (Fig. 2d) rather than being limited to a low concentration. The fixation of emulsion droplets in a metastable shape reveals the interfacial jamming of GO sheets (Fig. 2e). The emerged interfacial membrane after evaporating oil phase convincingly confirms the ultra-stability of interfaces that were sustained by GO/DDAB ion pairs (Fig. 2f and Supplementary Figs. 2 and 3)^42,43^.

More importantly, the emulsion that formed when c(GO) reaching 1.0 wt% (labeled as GO_1.0%_/DDAB) is clearly a stable gel (emulgel) with a significant stiffness (*G*’(*ω* = 1 s^−1^) = 2 × 10^4^ Pa in the linear viscoelastic range) (Fig. 2g) and a yield stress of 1.2 × 10^2^ Pa (Fig. 2h), which ensures continuous extrusion and rapid fixation into rigid filaments (Fig. 2e-inset). Compared with a pure aqueous GO dispersion with the same concentration [c(GO) = 1.0 wt%, labeled as GO_1.0%_ dispersion], the apparent viscosity and the plateau values of storage modulus (*G*′) and loss modulus (*G*″) increased by two orders of magnitude (Fig. 2h). This massive increase in viscosity and stiffness suggests that the viscoelastic behavior of the GO_1.0%_/DDAB emulgel is determined by the inherent structure of emulgel that involves packing, assembly, jamming of the GO in the continuous phase, droplet distribution, interfacial stiffness, and stability.

### Origin of high viscosity and moduli of emulgel

It has been shown that GO stacking and assembly affect the rheological behavior^44–46^. Under a high concentration (1 wt%), the short distance between the GO sheets strengthens their interactions (mainly hydrogen bonds at pH ∼2.3), limiting their mobility. In addition, GO jamming caused by the steric hindrance of large GO sheets also restrict the GO movements^45,47^. Both behaviors contribute to the considerable viscosity of the GO dispersion. Different from GO dispersion, a large number of interfaces are created in emulgel as the incorporation of emulsion droplets. The interfacial assembly promise to re-structure GO networks, jam droplets and GO sheets, and build large specific stiffer interfaces. Besides, the shear during homogenization also can alter the GO packing and interaction. Therefore, we investigate the effects of homogenization and interfacial assembly on rheological behavior, respectively.

After homogenization, the apparent viscosity of the GO_1.0%_ dispersion increased by 3.5 times, which was accompanied by an abnormal decrease of *G*′ and *G*″ by ca. 30% (Fig. 2h and Table S1). The result doesn’t obey the Cox-Merz rule^48^, as the large-scale movement of the sample is measured by the apparent viscosity function (*η*_app_ ($\dot \gamma$)), while the dynamic-mechanical test in the linear viscoelastic range is probing short-range interactions. To figure out the shear effects, we compared the GO packing in dispersion before and after homogenization by using polarized optical microscopy (POM) (Fig. 2i, j). The appeared long-range Schlieren texture and disappeared large-scale aggregations in the sheared GO_1.0%_ dispersion (Fig. 2j) indicate that the shear firstly helps to exfoliate the partially stacked GO sheets and then destroys networks formed by interacting GO sheets that trap the liquid in them (Fig. 2l, m). The exfoliated GO sheets form new hydrogen bonds and meanwhile produce more severe jamming due to the increased specific area, both of which render large amplitude motions more difficult, thus leading to the viscosity increase. We can imply that the highly increased viscosity of GO_1.0%_/DDAB emulgel is partially ascribed to the above shear effects during emulsification.

In contrast, dynamic-mechanical tests probe the local behavior only, and thus, the exfoliation increasing the moduli is a minor effect; while the destruction of the previously formed structure dominates the rheological behavior, leading to an overall decrease of moduli. These results also infer that the largely increased moduli *G*′ and *G*″ of GO emulgel (Fig. 2h) shall ascribe to the homogeneous droplet incorporation in the viscous GO dispersion.

Considering that the emulgel formation origins from GO and DDAB interfacial assembly, the effects of interfaces on rheological behavior are firstly investigated. The POM images of the GO_1.0%_/DDAB emulgel display a spherulite-like texture around the droplets (Fig. 2k), which indicates the parallel-banded structures of GO sheets around droplets^49,50^, as illustrated in Fig. 2n. Furthermore, a continuous membrane with a thickness of ∼28 nm forms at the O/W interface (Fig. 2o, p), suggesting a multiple-layer structure of the interfacial membrane. The GO self-assembly behavior was also investigated by direct observation of the assembly process (Supplementary Fig. 3) and a comparative study on GO/DDAB emulsions with different GO concentrations (Supplementary Figs. 4–6). All results indicate that the assembly of GO and DDAB leads to robust GO-rich interfaces rather than crosslinking GO into a gel. The stable GO/DDAB interfaces as the template to re-structure GO networks in narrow space between droplets, which results in more severe GO jamming. The jammed droplets and GO sheet itself lower the droplet movements and deformability and – as discussed before – increase the viscosity and stiffness of the emulgel^47,51^.

### 3D printing of Pickering emulgel inks

**Fig. 3:**
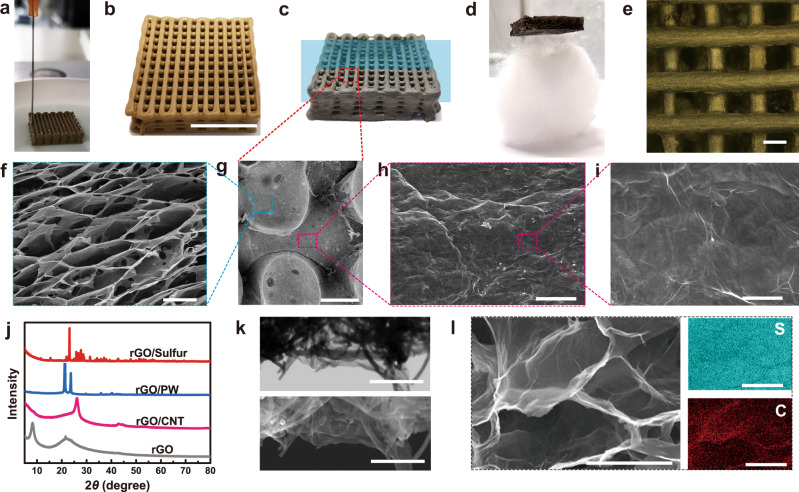
Morphology and characterization of nanomaterial matrices and their hybridization. **a**–**d** Images of **a** direct ink writing conduced on GO_1.0%_/DDAB-IB inks, **b** printed woodpile scaffolds sustained in air, **c** graphene aerogels, and **d** ultralight graphene aerogels with a density of ∼6 mg cm^−3^. **e** OM image of layer-by-layer stacking woodpile structure. **f**–**i** Scanning electron microscope (SEM) cross-sectional images (**f**, **g**) and surface images (**h**, **i**) of filaments in the printed woodpile. **j** X-ray diffractometry spectra of annealed 3D GO aerogels and 3D GO composites that include rGO/CNT and rGO/PW and rGO/Sulfur. **k** Transmission electron microscope (TEM) image and SEM image of rGO/CNT. **l** SEM image of GO/Sulfur and element mapping of S and C, respectively. Scale bars, 10 mm (**b**), 500 μm (**e**, **g**), 10 μm (**f**, **i**), 50 μm (**h**), 500 nm (**k**), and 5 μm (**l**).

The emulgel (GO/DDAB-IB) consisted of GO_1.0%_/DDAB and a volatile oil phase of iodobenzene (IB) was printed layer-by-layer into a woodpile structure to evaluate the printability and GO skeleton structure, as shown in Fig. 3a, b. After a series of treatments with in-situ crosslinking, chemical reduction, volatile IB removal, and freeze-drying (Supplementary Figs. 7–9), free-standing graphene aerogels were obtained (Figs. 3c, d). It is noted that freeze-drying results in a volumetric contraction of ∼27%, which should be evaluated before printing (Supplementary Fig. 10). The filaments in the woodpile structure maintain the cylindrical shapes (Fig. 3e, g), indicating the stability of the printed structures. The cross-sectional scanning electron microscope (SEM) images (Fig. 3f) of filaments show a highly interconnected honeycomb porous internal structure. The size of micropores at ~10–20 μm matches the original emulsion droplet size. Surface SEM images in Fig. 3h, i display that the filament surface is covered by a continuous membrane, indicating an anisotropic alignment of the GO sheets in the extrusion direction. Such a continuous membrane favors the preservation of agents in filaments.

The continuous GO phase of emulgel can also act as a carrier for other functional nanomaterials to improve the performance or equip with multiple functionalities, which has been demonstrated in monophasic GO inks^52^. However, the nanoparticle additives are limited to be water-soluble and negatively charged to ensure homogeneous hybridization. Here, carboxylate modified carbon nanotube (CNT) as a model nanoparticle additive was incorporated into a GO_1.0%_ dispersion to form a complex continuous nanomaterial phase, which produces a complex Pickering emulgel, as shown in Supplementary Fig. 11. The hybridization was confirmed by X-ray diffractometry (XRD) in Fig. 3j, X-ray photoelectron spectroscopy, Raman (Supplementary Figs. 8 and 9), and transmission Electron Microscope (TEM) images in Fig. 3k. The incorporation of CNT in the GO continuous phase promises the versatile hybridization capability of Pickering emulgel from the aqueous phase. More importantly, Pickering emulgel is advantageous over the reported monophasic nanomaterial inks in terms of its capability of simultaneous incorporation of water-soluble or non-water-soluble agents from both phases^33,52–55^.

Sulfur and liquid paraffin that represent the non-water-soluble solids and liquids, respectively, were encapsulated by GO_1.0%_/DDAB into emulgel, and both were printed into 3D structures, demonstrating the generality and versatility of this strategy (Supplementary Figs. 12 and 13). Compared with the XRD pattern of the annealed reduced GO (rGO) frameworks (Fig. 3j), each CNM-NC pattern shows the characteristic diffraction peaks of the corresponding additives, indicating the successful hybridizations. The interconnected graphene skeletons, homogeneous distribution on the graphene surface (Fig. 3l), and high loading sulfur content of 90% (Supplementary Fig. 12g) suggest the application of sulfur loading NCs in the fields of Li–S batteries. The stable storage of liquids in dried CNM-NCs provides promising applications in self-healing, energy storage, and catalysis.

### 3D printing of GO/PCM NCs

Composite PCMs are a class of typical CNM -NCs, which require a continuous porous matrix to encapsulate PCM to ensure stability, leakage-poof ability, and a tailorable thermal/electrical conductivity. Composite PCMs exhibit tremendous advantages in thermal management applications in batteries, supercapacitors, electronics, and solar energy harvesting and storage. However, their manufacturing technologies are limited to post-infiltrating, layer-by-layer casting, or hot pressing^15,18^, which produce limited bulk composite PCMs. Compared to the bulk composite PCMs, freeform engineered counterparts are undoubtedly more versatile and intelligent when integrated into devices, which would accelerate the development of internal thermal management or add the additional control on energy harvesting/conversion through regulating the structures^15,56,57^. Besides, as the upgrade of battery, supercapacitor, and electronic systems into three-dimension, developing 3D printing provides a promise for integrated multi-material printing to achieve self-thermal-management devices. Here, through 3D printing of GO/DDAB-paraffin wax (PW) emulgel ink, we enable freeform architecture of composite PCM and demonstrate the possibility of obtaining unique functions in 3D geometrical composite PCMs.

**Fig. 4:**
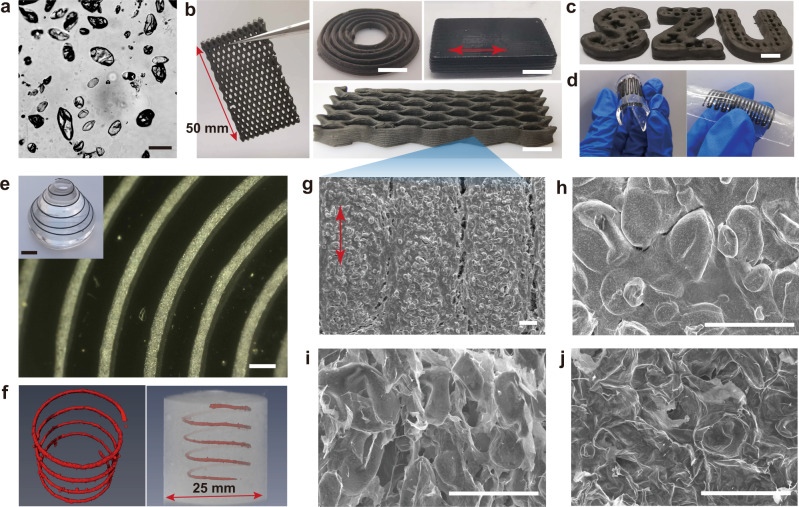
3D printing of PW@NCs. **a** OM image of GO_1.0%_/DDAB-PW emulsion droplets. **b**–**d** Optical images of various 3D-printed structures (**b**, **c**) and soft bent substrates with zigzag NCs embedded in **d**. **e** OM and optical images of the printed isometric spiral on the out-of-plane substrate. **f** Reconstructed 3D X-ray microcomputed tomography of the printed spiral in cement. **g**–**i** SEM surface (**g**, **h**) and cross-sectional images of PW@NC (**g**). **j** Cross-sectional SEM images of PW@NCs after annealing at 80 °C for 10 min. Scale bars, 100 μm (**a**), 5 mm (**b**, **c**, inset in e), 500 μm (**e**, **g**), 50 μm (**f**–**h**).

As shown in Fig. 4a, the GO/DDAB-PW emulsion was well dispersed in water and did not aggregate during PW solidification, which indicates the stable encapsulation by the GO/DDAB layers. CNT can further hybridize the GO/DDAB-PW into GO-CNT/DDAB-PW emulgel from the aqueous phase. Both inks were successfully printed into various programmed architectures, including a cubic block with aligned filaments, spiral cycle lines, fishing net structures, as well as a woodpile, zigzag (Fig. 4b), and SZU (Shenzhen University) shapes (Fig. 4c). The printed forms are labeled as PW@NC and CNT-PW@NC, respectively. After a series of treatments, including crosslinking, reduction, drying in the oven, and freeze-drying, the woodpile’s structural integrity remained intact and underwent a shrinkage of 19% for CNT-PW@NC (Supplementary Fig. 10). Besides, the woodpile structure shows excellent mechanical strength (Supplementary Fig. 14). Their delicate structures remained clearly when the compression strain reaches 20%, indicating that the printed PW@NCs can be further manufactured to improve their performance. Subsequently, the dried PW@NCs were also embedded in poly(dimethylsiloxane) PDMS to form a soft substrate, which could withstand extensive bending along or perpendicular to the printed lines (Fig. 4d).

Furthermore, the excellent printability of emulgel reveals 3D printing on out-of-plane substrates, which more strictly require high viscous inks, owing to easy slipping off the steep edges of curved surfaces (Fig. 4e). The constant diameter and isometric distance of the printed filaments indicate the accuracy of out-of-plane printing. Such a strategy broadens the freeform in designing applications involving composite PCMs to soft or out-of-plane devices.

The GO/DDAB-PW ink was also used to conduct the embedded 3D printing in cementitious materials, for example, the cement paste. Profiting by the high modulus of the inks, 3D spiral PW@NC was achieved, which precisely copied the program parameters, as shown by X-ray computed tomography images in Fig. 4f. The embedded printing of two-phase inks provides an option to program hydrophobic agents in the hydrophilic matrix, building thermal management systems in cement materials.

SEM images in Fig. 4g shows the aligned lines that consist of aggregated rGO/PW particles (Fig. 4f). The particles assemble into a continuous structure under the widespread coverage of the GO membrane (Fig. 4h and Supplementary Fig. 15). Notably, the stacking of solid rGO/PW particles leaves some space in the inner filaments (Fig. 4i), which adversely affects the thermal conductivity. Therefore, the printed structures were annealed at 80 °C to fix the particles together by softening the PW droplets (Supplementary Fig. 16). After this annealing treatment, a denser particle packing was achieved as revealed by surface and cross-sectional SEM images (Fig. 4j and Supplementary Fig. 15). All SEM testing of these samples was conducted without sputtering, demonstrating the good PW encapsulation by rGO.

### Emerged functions of 3D geometrical PW@NC

**Fig. 5:**
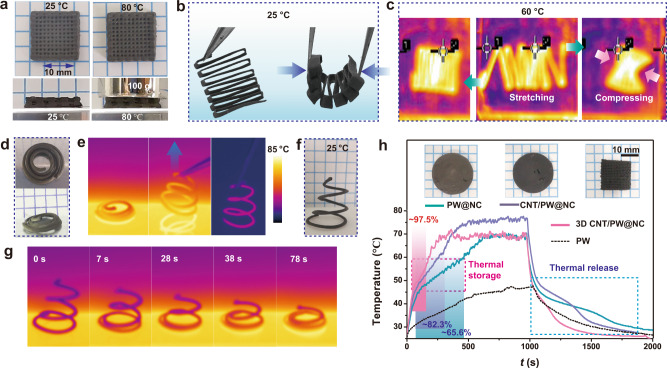
Performance of 3D-printed PW@NCs as phase-change material composites. **a** Top-view and side view of woodpile PW@NC at room temperature (RT) and 80 °C, respectively. Before and after treatment, the constant size and morphology indicate 3D-printed PW@NC stability even under 100 g loading. **b**, **c** 3D-pattern determined shape-change of PW@NC at room temperature (**b**) and shape-change above phase-change temperature (60 °C) (**c**). **d**–**g** 3D shape-change of CNT/PW@NC from in-plane to 3D spiral shape upon pulling (**d**, **e**) and complete reversion after re-heating (**g**). **f** CNT/PW@NC fixes the 3D shape at RT. The line spacing of the in-plane spiral is 2.0 mm. **h** Light-to-thermal conversion curves (**h**) and images (inset) of PW, 3D-printed PW@NCs, CNT/PW@NCs, and woodpile CNT/PW@NCs. The illumination was conducted under simulated sunlight (100 mW cm^−2^) for 1000 s.

The high-thermal energy storage density (Δ*H*_m_ > 152.1 J g^−1^) and completely retained Δ*H*_m_ after 100 heating and cooling cycles (Supplementary Figs. 17 and 18) demonstrate the excellent reversibility, efficiency, and thermal reliability of the printed structure. The leakage-proof test was then conducted on the woodpile PW@NC by heating at 80 °C to examine the shape stability. The well-maintained shape upon a load of 100 g (Fig. 5a) demonstrates the shape-stability, which endows PCM composite with excellent durability against phase changing of PW. Different from the traditional methods, 3D printing can generate shape-changing composite PCMs without liquid-leakage. The shape-changing ability favors the application of PCMs in flexible devices and allows the reversibly manufacturing between 2D and 3D structures. The engineering in real three-dimensions can overcome the shortcomings of DIW, which confines the printed filaments in layer-by-layer stacking. Such advancement facilitates the freeform and complex 3D engineering of composites PCMs. For example, a zigzag shape allowed for the bending of PW@NCs perpendicular to the plane at room temperature (RT) (Fig. 5b). When heated to 60 °C, the rigid structure became soft and could be stretched or compressed, as shown in Fig. 5c. Another example is the 2D-to-3D shape conversion of an in-plane isometric spiral to a 3D spiral upon pulling up at 80 °C (Fig. 5d, e and Supplementary Movie 1). The 3D spiral was fixed at room temperature and remained self-supporting, as shown in Fig. 5f. When placed the 3D spiral on a hot plate (80 °C), temperature conduction was observed along the filaments, followed by temperature-induced sagging back to an in-plane conformation in 78 s (Fig. 5g). This shape-changing ability favors a broadening for PCM application on flexible/out-of-plane 3D devices.

PW@NCs, as a blackbody, also exhibit photo-thermal conversion ability, thus shows excellent potential for application in photo-thermal energy conversion and storage. A high light-to-heat and energy storage efficiency (*η*) is an essential requirement for solar-electric thermal energy conversion and storage. The PW@NCs and CNT-PW@NCs were printed into bulk disk-like and woodpile, respectively (inset in Fig. 5h), and exposed to simulated solar light (100 mW cm^−2^). The calculated methods in the supplementary information estimated *η* to be ∼65.6 % for disk-shaped PW@NCs and ∼82.3% for CNT-PW@NCs, respectively. The difference indicates that the performance of the PW@NCs can be optimized by hybridization in a nanomaterial phase. Of critical importance, the *η* was further raised to ∼97.5% when irradiating the woodpile-patterned CNT-PW@NCs, being otherwise identical to the bulk CNT/PW@NCs sample. The massive improvement attributes to the increased harvesting efficiency of solar energy in hierarchical 3D structures. Therefore, 3D printing of a PW incorporated emulgel provides a valuable method to optimize the performance of light-thermal energy storage not only by hybridization but also by programming the intricate 3D topologies.

## Discussion

In summary, we have presented a versatile approach to preparing Pickering emulgel inks with all continuous nanomaterial phase and revealed the freeform architecture of nanocomposite with connected nanomaterial matrix (CNM-NC). The ultra-stable O/W interface formed by the interfacial assembly of oppositely charged nanomaterials and surfactants is fundamental to develop homogeneous Pickering emulgel. The GO jamming and interaction restrict droplet and GO itself mobility and deformability, which increases the viscosity and stiffness of the emulgel, yielding excellent printability. The Pickering emulgel inks allowed the versatile and homogeneous hybridization of water-soluble or non-water-soluble agents/particles from immiscible phases, demonstrating the capability of Pickering emulgel for mass customization and design flexibility. Enabled by this capability, we demonstrate the 3D printing fabrication of composite graphene/PCMs, as a proof concept of CNM-NC printing strategy. Beyond the remarkable thermophysical properties and shape-changing abilities, 3D-pattern-enhanced light-thermal conversion and storage efficiency (*η* ~ 97.5%) was also verified, which was higher than the best-reported value (95%). The 3D architecture of composite PCMs represents a significant step toward flexible, wearable, and 3D electronics. The facile and versatile nature of ion pair-reinforced emulgel ink provides a flexible platform for a wide array of multiphasic 3D frameworks that are required urgently in Li–S electrodes, seawater desalination devices, thermal management, electromagnetic shielding systems, and vascular self-healing systems.

## Methods

### Preparation of GO/DDAB emulgel and hybridized inks

Single-layer GO sheets with lateral dimensions of 2.0–5.0 μm were prepared from raw graphite powder (Aladdin) by a modified Hummers method (Supplementary Fig. 19). Aqueous GO dispersions with concentrations of 0.2, 0.3, 0.5, and 1.0 wt% were prepared and used as the continuous phase of emulsions. The GO-CNT hybridized dispersion was prepared by homogenizing a mixture of 1 mL of 10 wt% aqueous carboxylate-CNT (XFNANO Technology Co. Ltd.) and 9 mL of 1 wt% GO with a homogenizer (IKA T-10) at 9500 rpm for 1 min. Chloroform DDAB (98%, Aladdin) solution with a 1 wt% DDAB concentration was prepared initially and diluted with oil [iodobenzene (IB), liquid paraffin (LP), paraffin wax (PW), or saturated CS_2_ sulfur solution) to achieve an oil phase with DDAB of 0.1 wt%. The mixed oil phase (5 mL) was added to the aqueous phase (10 mL) and the mixture was emulsified with a homogenizer at 11,600 rpm for 3 min. The mixed PW oil phase, continuous phase, and dispersers were heated to 65 °C and kept warm during emulsification.

### 3D printing of Pickering emulgel inks

Emulgel inks were used to fabricate a 3D structure using a robotic deposition device (TH-206H, Tianhao Technic, China). Three-dimensional models of various constructs were programmed using the provided software, which translates this information into a G-code to coordinate the motion of the pneumatic syringe. Needles with diameters of 160, 250, and 330 μm were used. All 3D NCs were printed layer-by-layer with a constant extrusion pressure of 0.5–1.5 bar, which depends on the nozzle size and ink viscosity. Based on primary optimization, the moving speed of the nozzle was 10 mm s^−1^ and the initial nozzle height from the substrate was approximately 0.6 mm to help in the precise tracking the path of the nozzle.

The as-printed 3D structures were immersed in an aqueous crosslinker aqueous solution [Ethylenediamine (EDA)] for 30 min to ensure thorough crosslinking and were reduced in hydrazine hydrate vapor at 60 °C for 24 h. When the oil phase was a volatile solvent (IB), a solvent-exchange process with water was performed after crosslinking. The reduction extent was characterized by XPS and Raman spectra (Supplementary Figs. 8 and 9). The 3D NCs were washed with DI water for 3 times under repeat centrifuge to remove the residual EDA, hydrazine hydrate, and DDAB, followed by freeze-drying (in liquid nitrogen) for 12 h to obtain dried 3D NC. When the oil phase was PW, 3D NCs underwent oven-drying at 50 °C for 3 h and freeze-drying for 12 h.

### Characterization

Ink rheological characteristics were measured with a rheometer (TA, Discovery HR 30). An oscillatory logarithmic stress sweep (10^−1^−10^4^ Pa) was adopted to measure the moduli at a constant frequency of 1 Hz, and the apparent viscosity was recorded with a strain sweep at shear rates from 0.01 to 200 s^−1^. All experiments were conducted at 25 °C. Optical microscopy (OM) of the emulsions before and after dilution and polarized optical microscopy (POM) images of the GO dispersion and GO emulgel were captured by using an Eclipse TS100 optical microscope (Nikon, Japan). Confocal laser scanning microscopy (CLSM) images of the GO emulgel were acquired by using a TCS SP5 II laser scanning microscope (Leica, Germany) with Nile red as the fluorescent marker for the oil phase. Notably, the characterizations of rheology, OM, and CLSM were conducted on GO/DDAB-IB emulgel. The morphology of various 3D-printed NCs was examined by SU-70 high-resolution microscopy (Hitachi, Japan) at an accelerating voltage of 10 kV, and a high-resolution scanning electron microscope (FEI APREO S(A5-112)) at an accelerating voltage of 30 kV. XRD patterns were obtained by using an Ultima IV diffractometer. The latent heat and phase-change temperature of the samples was characterized by using a differential scanning calorimeter (DSC, TA Q20, USA) at a heating/cooling rate of 10 °C min^−1^ in a highly purified nitrogen atmosphere. Thermal infrared images were recorded with an infrared camera (TiS65, Fluke, USA). The compression test is applied on 3D-printed PW@NCs [c(GO) = 1.0 wt%)] framework by using a testing machine (SANS, CMT4204).

A light-to-thermal energy conversion test was carried out under simulated sunlight (100 mW cm^−2^) provided by a solar simulator (Zolix ss150). A paperless recorder with thermocouples was used to record the temperature.

## Supplementary information

Supplementary Information

Description of Additional Supplementary Files

Supplementary Movie 1

## Data Availability

All data generated in this study can be found in this published article or Supplementary Data.

## Code availability

The custom codes used in this study for 3D printing are available from the corresponding author upon reasonable request.

## References

[1] Kim JH (2016). Three-dimensional printing of highly conductive carbon nanotube microarchitectures with fluid ink. ACS Nano.

[2] Yang (2019). Electrically assisted 3D printing of nacre-inspired structures with self-sensing capability. Sci. Adv..

[3] Kotz F (2017). Three-dimensional printing of transparent fused silica glass. Nature.

[4] Gao T (2019). 3D printing of tunable energy storage devices with both high areal and volumetric energy densities. Adv. Energy Mater..

[5] Bodkhe S, Turcot G, Gosselin FP, Therriault D (2017). One-step solvent evaporation-assisted 3D printing of piezoelectric PVDF nanocomposite structures. ACS Appl. Mater. Interfaces.

[6] Zhang M (2020). Microribbons composed of directionally self-assembled nanoflakes as highly stretchable ionic neural electrodes. Proc. Natl Acad. Sci. USA.

[7] Guiney LM (2018). Three-dimensional printing of cytocompatible, thermally conductive hexagonal boron nitride nanocomposites. Nano Lett..

[8] Bozuyuk U (2018). Light-triggered drug release from 3D-printed magnetic chitosan microswimmers. ACS Nano.

[9] Farahani RD, Dube M, Therriault D (2016). Three-Dimensional printing of multifunctional nanocomposites: manufacturing techniques and applications. Adv. Mater..

[10] Truby RL, Lewis JA (2016). Printing soft matter in three dimensions. Nature.

[11] Zhou LY, Fu J, He Y (2020). A review of 3D printing technologies for soft polymer materials. Adv. Funct. Mater..

[12] Müller LAE, Zimmermann T, Nyström G, Burgert I, Siqueira G (2020). Mechanical properties tailoring of 3D printed photoresponsive nanocellulose composites. Adv. Funct. Mater..

[13] Dermanaki Farahani R, Dubé M (2018). Printing polymer nanocomposites and composites in three dimensions. Adv. Eng. Mater..

[14] Fu K, Yao Y, Dai J, Hu L (2017). Progress in 3D printing of carbon materials for energy-related applications. Adv. Mater..

[15] Yuan K (2019). Engineering the thermal conductivity of functional phase‐change materials for heat energy conversion, storage, and utilization. Adv. Funct. Mater..

[16] Gao T (2017). Three-dimensional printed thermal regulation textiles. ACS Nano.

[17] Liang Z (2019). General, vertical, three-dimensional printing of two-dimensional materials with multiscale alignment. ACS Nano.

[18] Yang J (2019). High-performance composite phase change materials for energy conversion based on macroscopically three-dimensional structural materials. Mater. Horiz..

[19] Wu S (2019). High‐performance thermally conductive phase change composites by large‐size oriented graphite sheets for scalable thermal energy harvesting. Adv. Mater..

[20] Shen K, Mei H, Li B, Ding J, Yang S (2018). 3D printing sulfur copolymer-graphene architectures for Li-S batteries. Adv. Energy Mater..

[21] Han J, Du G, Gao W, Bai H (2019). An anisotropically high thermal conductive boron nitride/epoxy composite based on nacre‐mimetic 3D network. Adv. Funct. Mater..

[22] Lewis JA (2006). Direct ink writing of 3D functional materials. Adv. Funct. Mater..

[23] Barg S (2014). Mesoscale assembly of chemically modified graphene into complex cellular networks. Nat. Commun..

[24] Sommer MR (2017). 3D printing of concentrated emulsions into multiphase biocompatible soft materials. Soft Matter.

[25] Huan S, Ajdary R, Bai L, Klar V, Rojas OJ (2019). Low solids emulsion gels based on nanocellulose for 3D-printing. Biomacromolecules.

[26] Huan S (2019). Two‐phase emulgels for direct ink writing of skin‐bearing architectures. Adv. Funct. Mater..

[27] Schrade A, Landfester K, Ziener U (2013). Pickering-type stabilized nanoparticles by heterophase polymerization. Chem. Soc. Rev..

[28] Dinkgreve M, Velikov KP, Bonn D (2016). Stability of LAPONITE(R)-stabilized high internal phase Pickering emulsions under shear. Phys. Chem. Chem. Phys..

[29] Pang B (2020). High-internal-phase pickering emulsions stabilized by polymeric dialdehyde cellulose-based nanoparticles. ACS Sustain. Chem. Eng.

[30] Kim K (2017). Processable high internal phase Pickering emulsions using depletion attraction. Nat. Commun..

[31] Zhu Y (2020). High internal phase oil-in-water pickering emulsions stabilized by chitin nanofibrils: 3D structuring and solid foam. ACS Appl. Mater. Interfaces.

[32] Siqueira G (2017). Cellulose nanocrystal inks for 3D printing of textured cellular architectures. Adv. Funct. Mater..

[33] Zhu C (2015). Highly compressible 3D periodic graphene aerogel microlattices. Nat. Commun..

[34] Zhang CJ (2019). Additive-free MXene inks and direct printing of micro-supercapacitors. Nat. Commun..

[35] Li X, Li H, Fan X, Shi X, Liang J (2020). 3D‐printed stretchable micro‐supercapacitor with remarkable areal performance. Adv. Energy Mater..

[36] Creighton MA, Ohata Y, Miyawaki J, Bose A, Hurt RH (2014). Two-dimensional materials as emulsion stabilizers: interfacial thermodynamics and molecular barrier properties. Langmuir.

[37] Kim J (2010). Graphene oxide sheets at interfaces. J. Am. Chem. Soc..

[38] Shi S, Russell TP (2018). Nanoparticle assembly at liquid-liquid interfaces: from the nanoscale to mesoscale. Adv. Mater..

[39] Sun Z, Feng T, Russell TP (2013). Assembly of graphene oxide at water/oil interfaces: tessellated nanotiles. Langmuir.

[40] Zhang Y (2019). Salt-triggered release of hydrophobic agents from polyelectrolyte capsules generated via one-step interfacial multilevel and multicomponent assembly. ACS Appl. Mater. Interfaces.

[41] Cui M, Emrick T, Russell TP (2013). Stabilizing liquid drops in nonequilibrium shapes by the interfacial jamming of nanoparticles. Science.

[42] Liu X (2017). Liquid tubule formation and stabilization using cellulose nanocrystal surfactants. Angew. Chem. Int. Ed. Engl..

[43] Huang C (2016). Structured liquids with pH-triggered reconfigurability. Adv. Mater..

[44] Liu Y, Xu Z, Gao W, Cheng Z, Gao C (2017). Graphene and other 2D colloids: liquid crystals and macroscopic fibers. Adv. Mater..

[45] Naficy S (2014). Graphene oxide dispersions: tuning rheology to enable fabrication. Mater. Horiz..

[46] Aboutalebi SH, Gudarzi MM, Zheng QB, Kim J-K (2011). Spontaneous formation of liquid crystals in ultralarge graphene oxide dispersions. Adv. Funct. Mater..

[47] Chen DTN (2010). Rheology of soft materials. Annu. Rev. Condens. Matter Phys..

[48] Cox WP, Merz EH (1958). Correlation of dynamic and steady flow viscosities. J. Polym. Sci..

[49] Yang H (2018). Reconstruction of inherent graphene oxide liquid crystals for large-scale fabrication of structure-intact graphene aerogel bulk toward practical applications. ACS Nano.

[50] Wan W (2015). Graphene oxide liquid crystal Pickering emulsions and their assemblies. Carbon.

[51] Barnes HA (1994). Rheology of emulsions— a review. Colloids Surf. A: Physicochemical Eng. Asp..

[52] Tang X (2018). Generalized 3D printing of graphene-based mixed-dimensional hybrid aerogels. ACS Nano.

[53] Dong L (2018). A non-dispersion strategy for large-scale production of ultra-high concentration graphene slurries in water. Nat. Commun..

[54] Jiang Y (2018). Direct 3D printing of ultralight graphene oxide aerogel microlattices. Adv. Funct. Mater..

[55] Cao D (2019). 3D printed high-performance lithium metal microbatteries enabled by nanocellulose. Adv. Mater..

[56] Mohammadian SK, He Y-L, Zhang Y (2015). Internal cooling of a lithium-ion battery using electrolyte as coolant through microchannels embedded inside the electrodes. J. Power Sources.

[57] Wu W (2019). A critical review of battery thermal performance and liquid based battery thermal management. Energy Convers. Manag..

