# Supplementary material for: Interfacial jamming reinforced Pickering emulgel for arbitrary architected nanocomposite with connected nanomaterial matrix
Source: Nat Commun. 2021 Jan 4;12:111. doi: 10.1038/s41467-020-20299-6 (PMC7782697; doi:10.1038/s41467-020-20299-6)
Supplement: Supplementary file 1 — Supplementary Information [file 41467_2020_20299_MOESM1_ESM.pdf]

Supplementary information

Interfacial Jamming Reinforced Pickering Emulgel for Arbitrary Architected Nanocomposite with Connected Nanomaterial Matrix

Yuanyuan Zhang[1,2], Guangming Zhu[2], Biqin Dong[1], Feng Wang[3], Jiaoning Tang[2], Florian J. Stadler,[2] Guanghui Yang,[2] Shuxian Hong[1], Feng Xing[1*]

[1]Department of Civil and Transportation Engineering, Guangdong Province Key Laboratory of Durability for Marine Civil Engineering, Shenzhen 518060, P. R. China.

[2]College of Materials Science and Engineering, Shenzhen University, Shenzhen 518071, P. R. China.

[3]Department of Materials Science and Engineering, City University of Hong Kong, 83 Tat Chee Avenue, Hong Kong SAR, P. R. China.

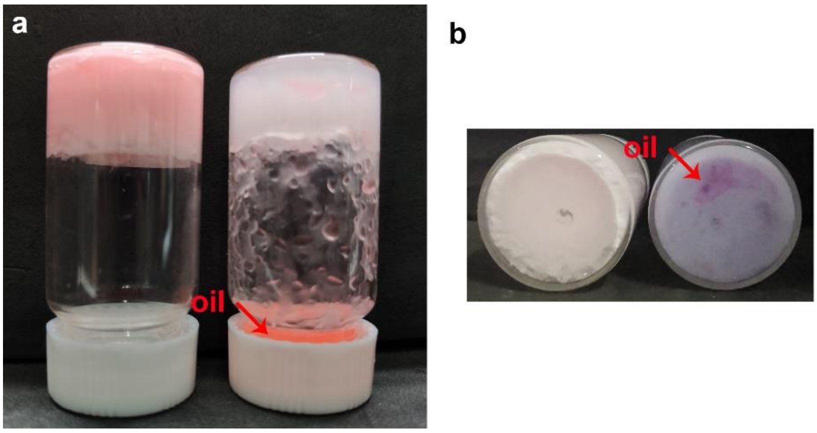

Supplementary Fig. 1 Comparative study on emulsification when using CNF [c(CNF) = 4 wt%] and laponite [c(laponite) = 4 wt%] as the continuous phase and liquid paraffin with/without DDAB dissolved in as the oil phase. The absence of DDAB results in the filature of emulsification.

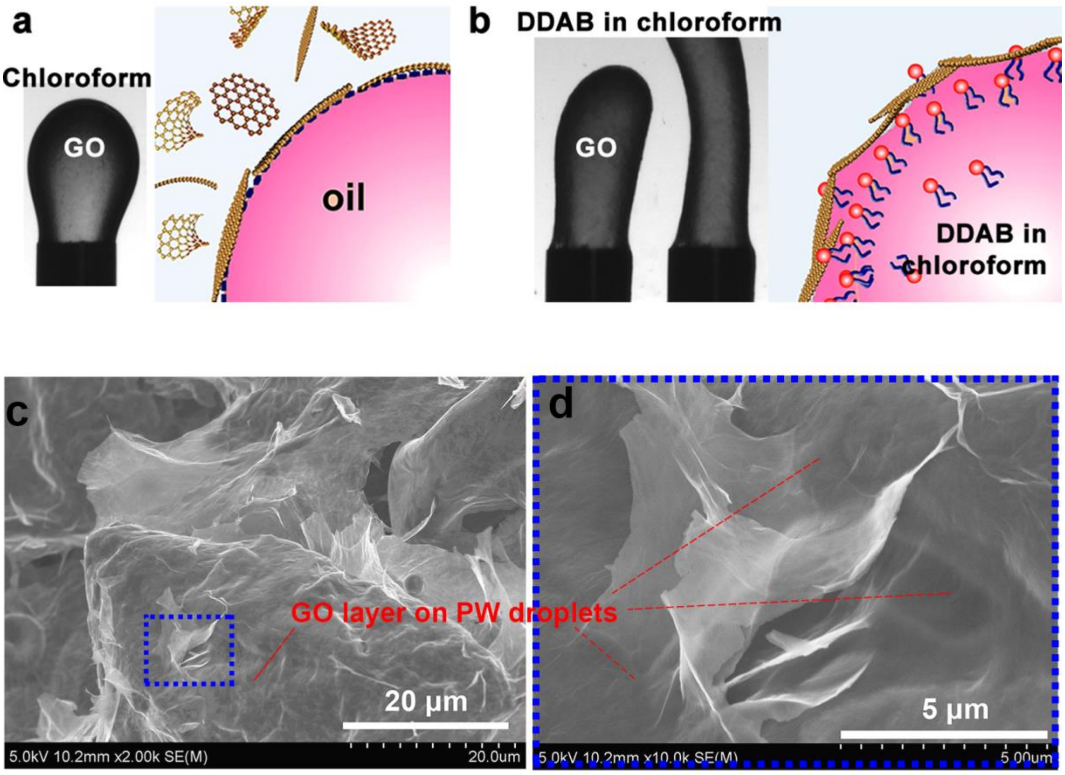

Supplementary Fig. 2 (a, b) Photo images of GO droplets when extruding aqueous $GO_{1.0\%}$

dispersion in (a) chloroform and (b) chloroform DDAB solution [c(DDAB) = 0.1 wt%] respectively. Differently, the droplet in chloroform only displays a volume increase rather than elongation. The droplet in chloroform DDAB solution elongates as the continuous extrusion, indicating the formation of an ultrastable interface. That is owing to the GO interfacial jamming at O/W interface induced by DDAB. The schematics in (a) and (b) illustrate the interfacial assemblies of GO at O/W interface with/without DDAB. (c, d) SEM images of GO/DDAB emulsion droplets with solid core (paraffin wax). The droplets were covered by a typical wrinkled graphene layer, which ensures the direct morphology observation under SEM without sputtering the gold layer. All the characterizations indicate the formation of GO interfacial jamming layer.

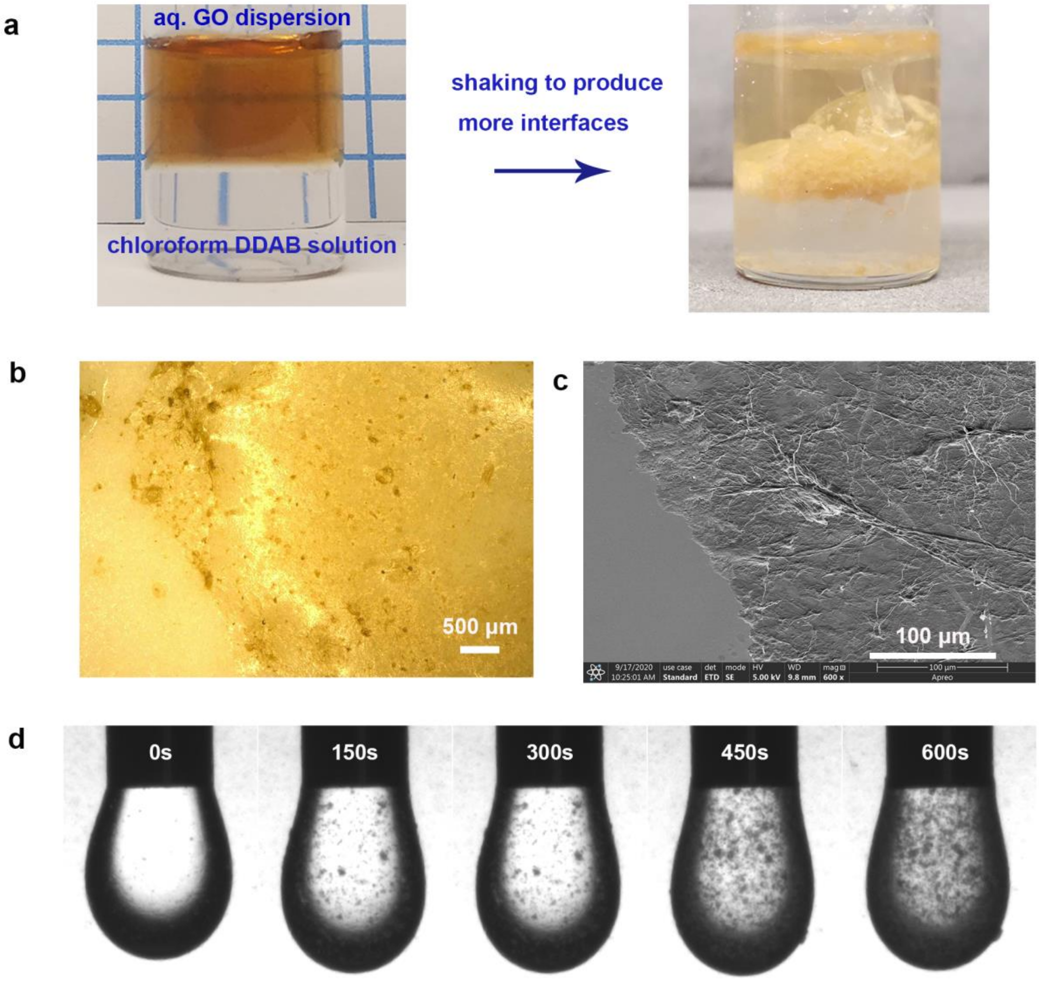

Supplementary Fig. 3 (a) To facilitate the characterization of the interfacial membrane, large-scale membranes were achieved by applying aqueous GO dispersion (0.05 wt%) on chloroform DDAB solution (0.1 wt%). The interfacial membrane generates immediately at oil/water interface. To keep the complete growth, the membrane was preserved at interface for more than 15 minutes before observation under various microscopes. During the growing process, no aggregates were produced in both phases, demonstrating the confined interfacial assembly behavior of GO and DDAB. Shaking the mixture made more interfaces, allowing more membranes formed, as shown by image (a). The optical microscope image of the interfacial membrane was achieved after removing the upper GO solution. The membrane was continuous but brittle (b) owing to the thin thickness as determined by AFM characterization (Figure 2h). (c) SEM image displays the typical wrinkled structure of GO membrane. (d) The optical images of DDAB/chloroform droplets at different preserving time

in aqueous GO dispersion [c(GO) = 0.05 wt%)]. Obviously, GO sheets were adsorbed on the droplet surface and no aggregates formed around droplets. We use aqueous GO dispersion with a concentration of 0.05 wt% as the outer phase to reduce the transparency, thereby ensure the clear observation of interface during assembly.

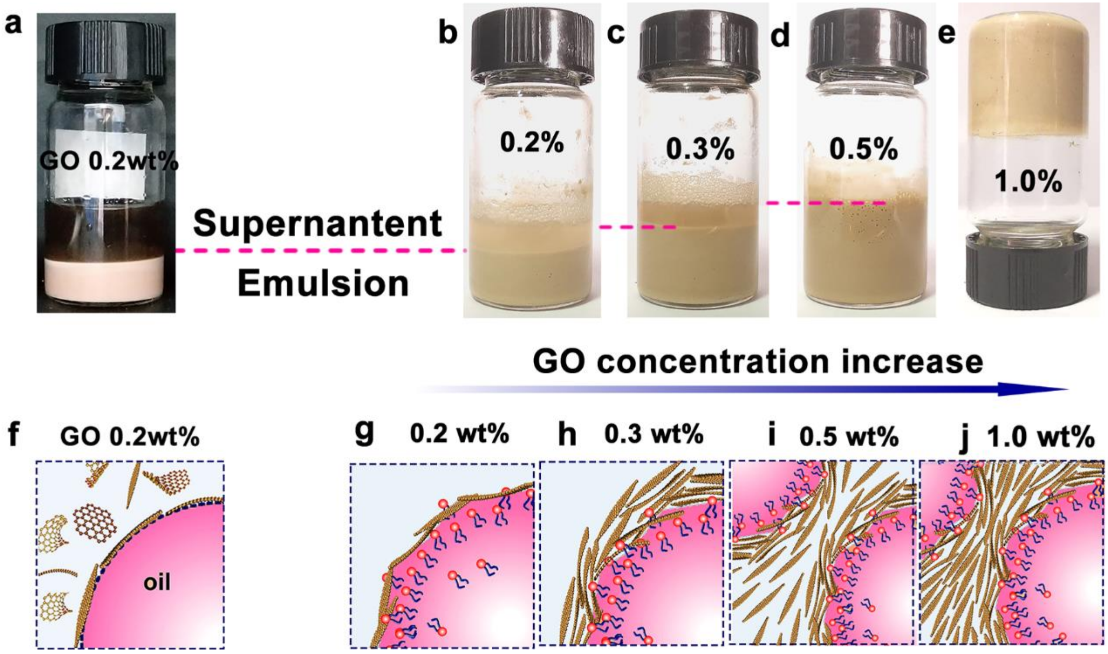

Supplementary Fig. 4 (a-e) Optical images of $GO_{0.2\%}$/IB emulsion (a) and (b-e) GO_x/DDAB-IB emulsion (GO concentration x = 0.2 wt%, 0.3 wt%, 0.5 wt%, 1.0 wt%). (f-j) Schematic illustrations of GO assembly on O/W interfaces in $GO_{0.2\%}$/IB emulsion (f) and GO_x/DDAB-IB emulsion (g-j).

The comparative studies on the supernatant and the droplet morphologies of GO emulsions with different GO concentration primarily disclose GO/DDAB emulgel structure (Supplementary Fig. 4 and Supplementary Fig. 5). Unlike the situation that a large amount of GO sheets are left in the supernatant of $GO_{0.2\%}$ emulsions (Supplementary Fig. 4a); the light color of supernatant in $GO_{0.2\%}$/DDAB emulsion indicates almost all of GO absorbed on the

O/W interfaces (Supplementary Fig. 4b, 4g). This result suggests that more GO jam onto the oil/water interfaces owing to the interfacial assembly with DDAB. As the GO concentration increase to 0.3 wt%, the supernatant of $GO_{0.3\%}$/DDAB emulsion become slightly dark (Supplementary Fig. 4c), indicating the excess GO in the continuous phase and saturated adsorption of GO sheets on the surface of oil droplets, as illustrated in Supplementary Fig. 4h. This result further demonstrates the limited DDAB diffusion toward the aqueous phase. The exceeded GO cannot adsorb on the interfaces and instead preserve in the supernatant.

Further increasing GO concentration over 0.5 wt%, no supernatant is observed, demonstrating that the surplus GO is preserved among the droplets (Supplementary Fig. 4d, 4i). The GO preservation no longer relies on the interaction with DDAB and is instead based on hydrogen-bonding interaction between GO sheets. As GO concentration increases to 1 wt%, the droplet size decreases and the anisotropy increases (Supplementary Fig. 5), which means more oil/water interfaces are created. The high specific interface area subdivides the continuous phase into more narrow spaces (between droplets), which further confines the GO packing and strengthens the GO jamming. The more severe GO jamming in a more confined limited space further lowers the droplet movements and deformability and – as discussed before – increases the viscosity and stiffness of the emulgel. Therefore, compare to $GO_{0.5\%}$/DDAB-IB emulgel, the platform storage modulus (G'), loss modulus (G") of $GO_{1.0\%}$/DDAB-IB emulgel increased by approximately 6.5 times of magnitude, and viscosity increased by 5 times, as shown in Supplementary Fig. 6 and Supplementary Table 1.

In addition, GO sheets jammed on the interfaces to form stiff membranes, which also contribute to the high moduli of emulgel.

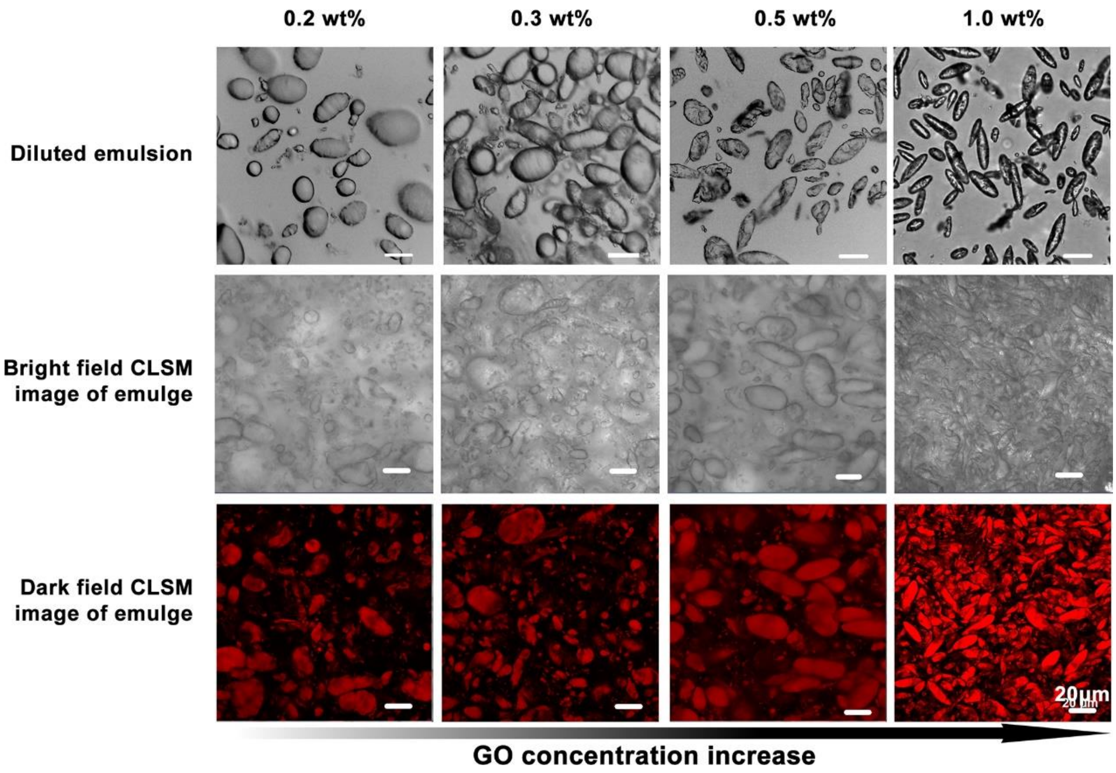

Supplementary Fig. 5 The optical microscope images of diluted GO_x/DDAB (x = 0, 0.2 wt%, 0.3 wt%, 0.5 wt%, 1.0 wt%) emulsions with and bright-field and dark-field CLSM images of GO_x/DDAB emulsions after removing the supernatant. The anisotropic emulsion droplets keep stable after dilution, demonstrating the ultra stability of interface. Scale bars, 20 µm.

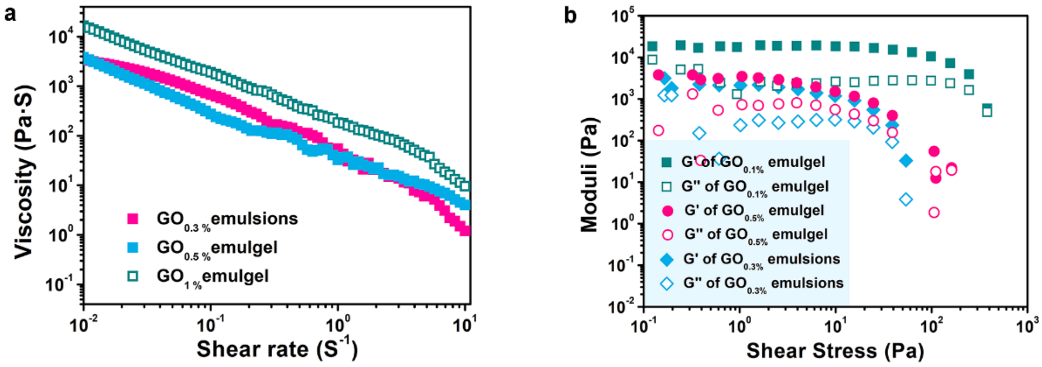

Supplementary Fig. 6 Rheological behaviors of GO_x/DDAB emulsions (x = 0.3 wt%, 0.5 wt%, 1.0 wt%) after removing the supernatant. G´ and G´´ respectively represent the storage modulus and loss modulus.

Supplementary Table 1. The plateau values of *G´*, *G´´*and apparent viscosity of $GO_{1.0\%}$ dispersion with and without sheared and GO_x/DDAB emulsions (*x* = 0.3 wt%, 0.5 wt%, 1.0 wt%), respectively. "Emulgel" is shorted into E in the table.

	**$GO_{1.0\%}$-E**	**$GO_{0.5\%}$-E**	**$GO_{0.3\%}$-E**	**$GO_{1.0\%}$**	**Sheared $GO_{1.0\%}$**
**G'(ω = 1rad/s)/Pa**	19462	3205	2131	298	209
**G'' (ω = 1rad/s)/Pa**	2634	755	311	102	50
**$\eta_{app}(\dot{\gamma}$ = 0.01 s^{-1})/Pa· s^{-1}**	23972.4	6372.8	4330.5	242	952

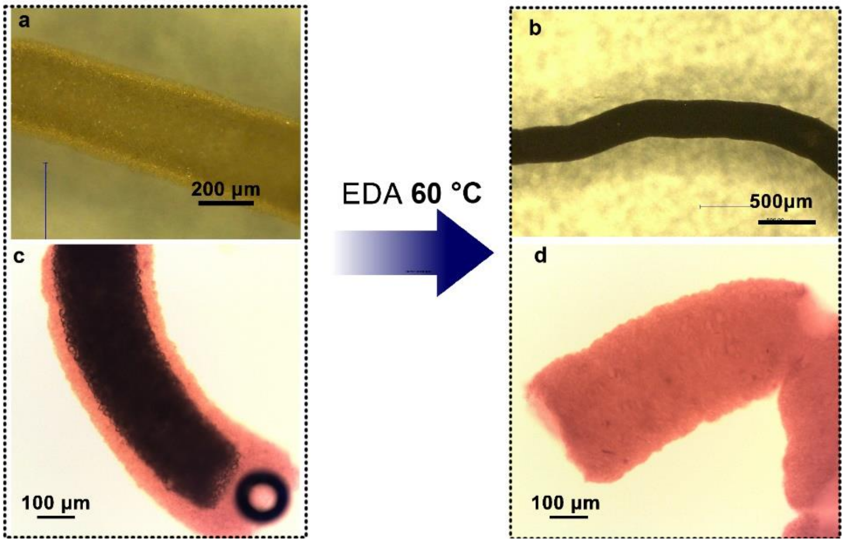

Supplementary Fig. 7 The stereomicroscope images (a, b) and optical microscope (OM) image (c, d) of the extruded filaments in EDA. After heating at 80 °C for 4 hours, the filaments turned black, demonstrating crosslinking and reduction. Simultaneously, the solvent was removed and porous frameworks were observed under the transmission channel, indicating

the stability of the crosslinked nanomaterial networks.

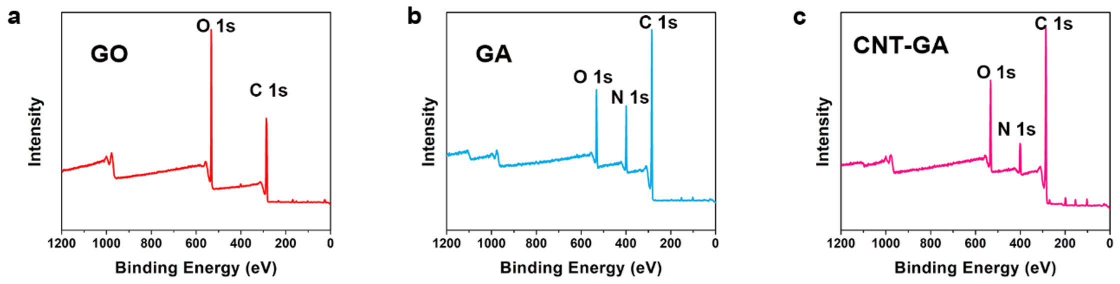

Supplementary Fig. 8 XPS survey scan of (a) GO, (b) graphene aerogel (GA), and CNT-GA, respectively.

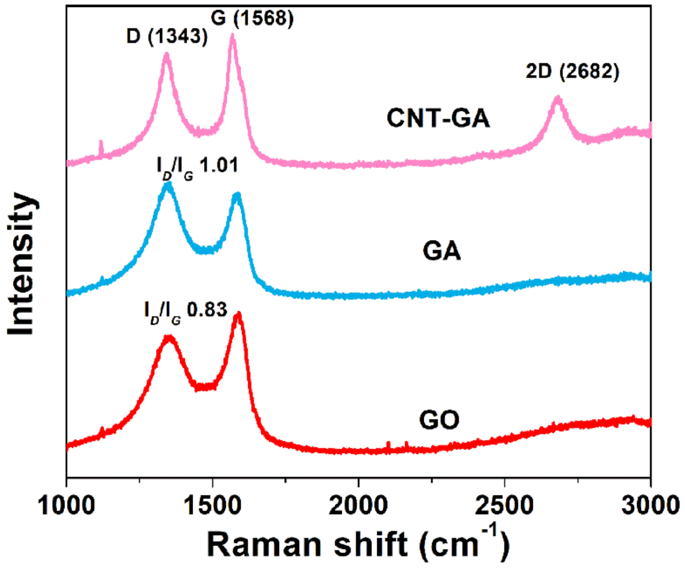

Supplementary Fig. 9 Raman spectra of GO, GA, and CNT-GA, respectively. The Raman spectra of GO and GA samples both display two absorbance regions: D-band near 1340 cm^{-1} (C–C, disordered graphite structure) and G-band near 1590 cm^{-1} (sp^2-hybridized carbon). The intensity ratio of D/G (I_D/I_G), representing the relationship between disorderly carbon and orderly graphitized carbon, increased from 0.83 to 1.01, confirming the recrystallization of graphene.

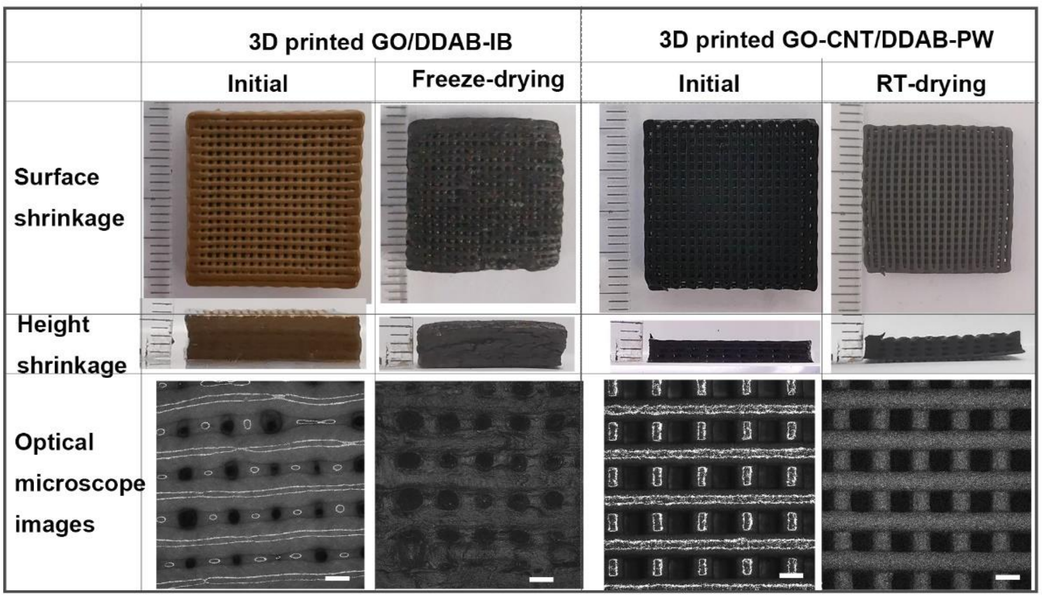

Supplementary Fig. 10 Shape retention ability of printed woodpile GO/DDAB-IB and GO-CNT/DDAB-PW frameworks. The top-view and side-view optical images and optical microscope images of frameworks before and after drying are compared. Scale bars, 500 µm.

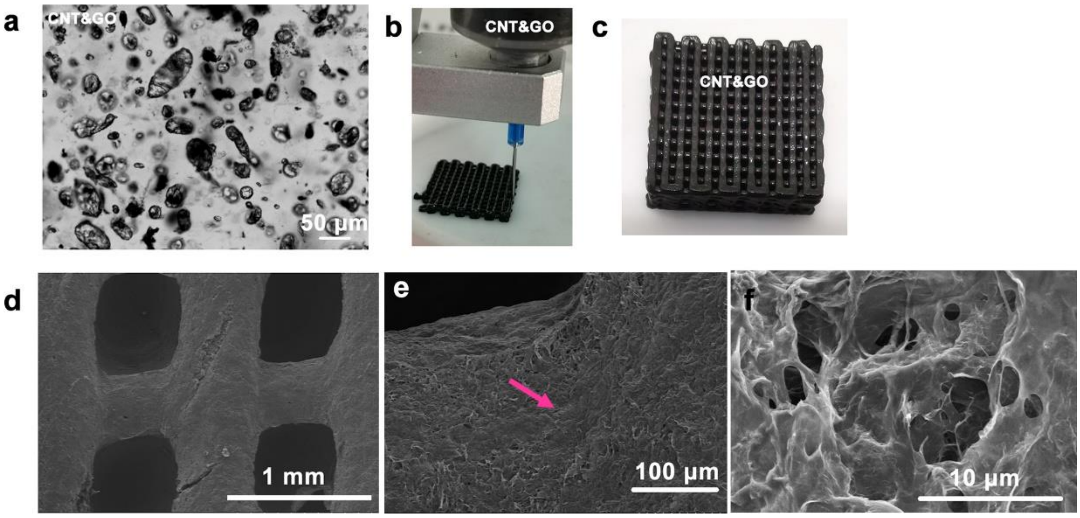

Supplementary Fig. 11 (a) OM images of (a) GO-$CNT_{1.0\%}$/DDAB-IB emulsions. (b, c) photo images of (b) direct ink writing of GO-$CNT_{1.0\%}$/DDAB-IB ink and (c) the printed woodpile structures. (d-f) SEM images of the woodpile aerogel. SEM image in (e) exhibited the tight junction between the orthogonal filaments.

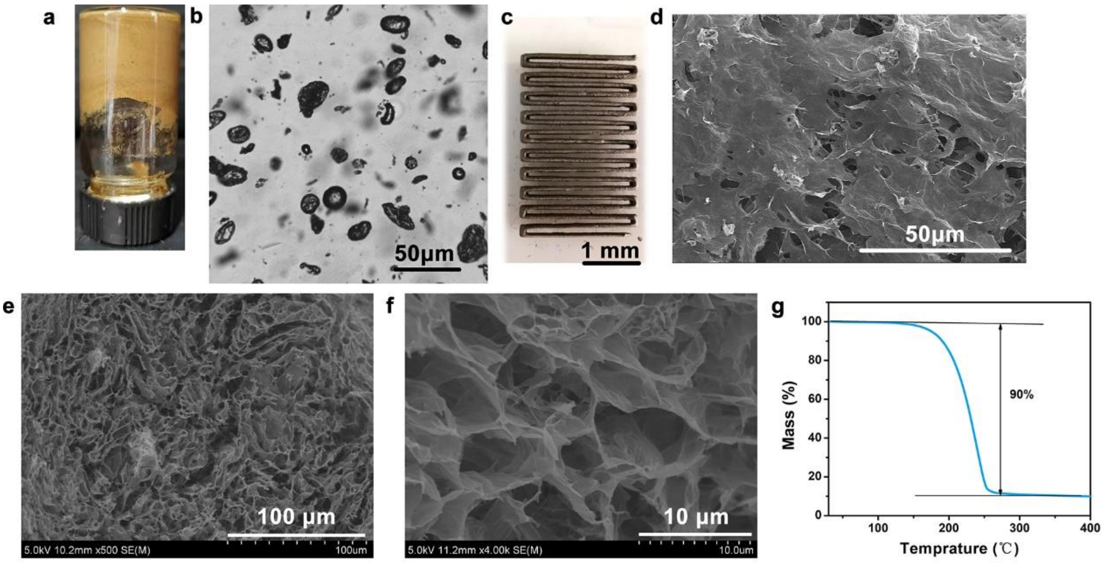

Supplementary Fig. 12 The characterizations on $GO_{1.0\%}$/DDAB-CS_2-sulfur emulsions and sulfur-NCs. (a) Photo image of the highly viscous $GO_{1.0\%}$/DDAB-CS_2-sulfur emulgel. (b) OM images of $GO_{1.0\%}$/DDAB-CS_2-sulfur emulsions. (c) Zigzag-shaped sulfur-NCs with a dimension of 20 mm × 30 mm. (d-e) SEM image of (d) the filament surface and (e, f) cross-sections. No large-sized crystals are observed from both the surface and cross-section, indicating the homogeneous crystallization of sulfur in graphene frameworks. (g) TGA curves of sulfur-NCs.

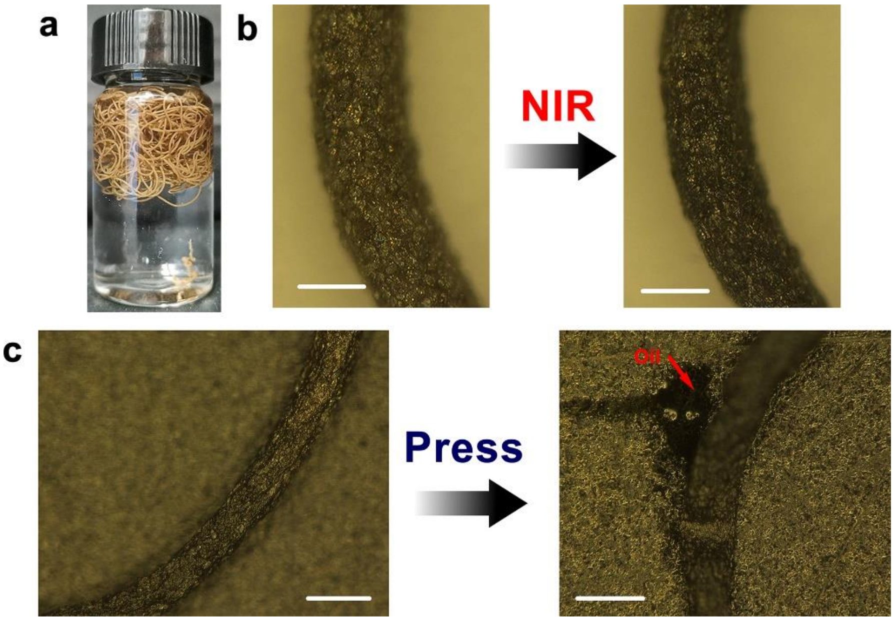

Supplementary Fig. 13 (a) The optical image of $GO_{1.0\%}$/DDAB-Liquid paraffin (LP) filaments that formed by extruding emulgel in Ethylenediamine. (b) Under the irradiation of NIR (100 w), no LPs leaks from the filaments, demonstrating the preserving ability of filaments. The LP was well preserved in the dried filaments and leak-off under squeeze. Scale bar, 250 µm.

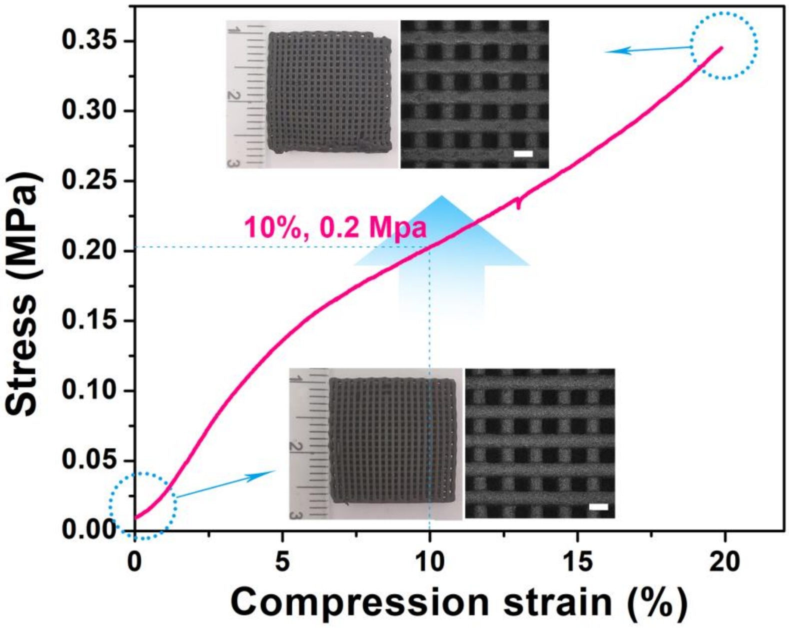

Supplementary Fig. 14 Compression tests on printed PW@NCs [c(GO) = 1.0 wt%)] woodpile frameworks. Insets are the optical images of woodpile frameworks before and after compression for 20% strain. When the compression strain is 10%, the stress reaches 0.2 MPa. When the compression strain reaches 20%, the printed structure was pressed flat but remain

integrated. These results indicate an excellent mechanical strength of PW@NCs. Scale bar, 500 μm.

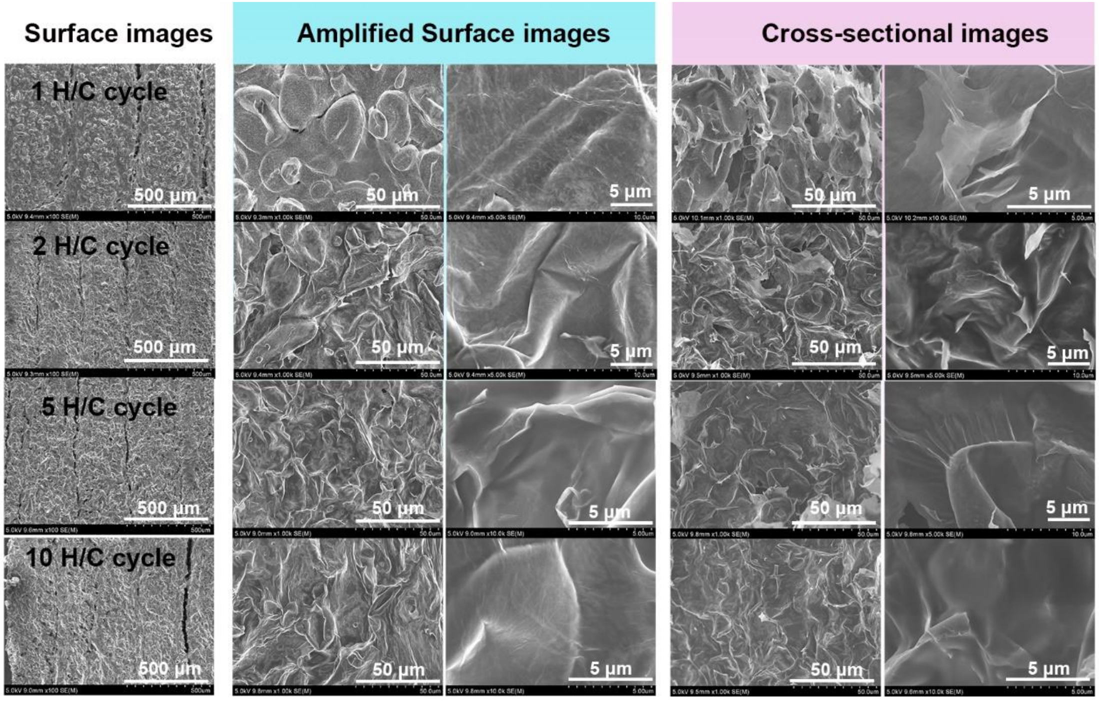

Supplementary Fig. 15 SEM images of PW@NCs withstand the heating/cooling (H/C) for different cycles. As the increase of H/C cycles, the graphene/PW particles change their shape to fill up the intervals in NCs. The typically wrinkled graphene morphology on filaments remained clear after 10 heating/cooling cycles, which confirms the shape-stability of the PW@NCs.

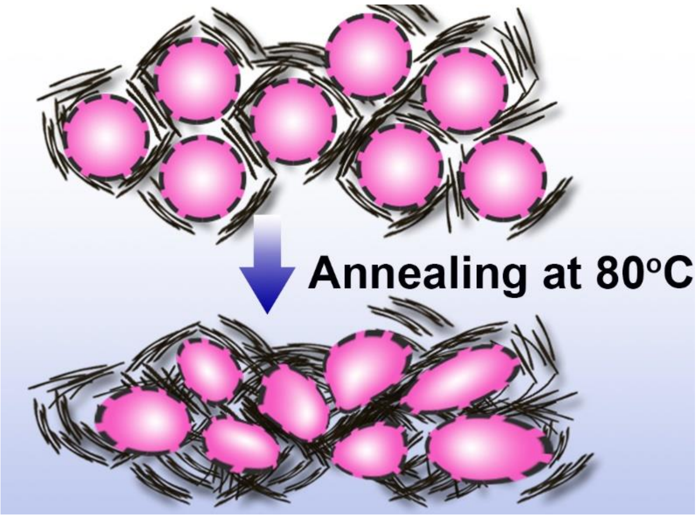

Supplementary Fig. 16 Schematic illustration of the structure change after annealing.

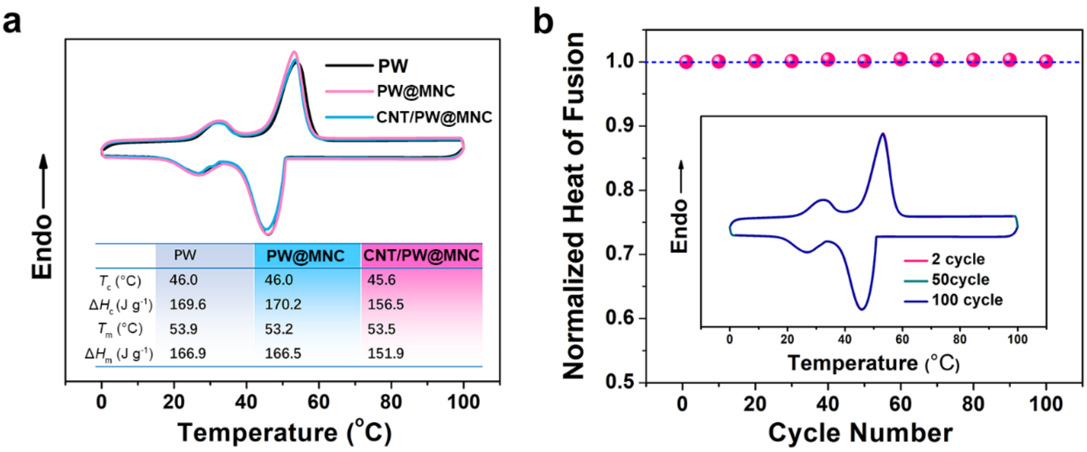

Supplementary Fig. 17 (a) DSC heating and cooling curves of pure PW, PW@NCs, CNT/PW@NCs. (b) The obtained mass-specific heat of fusion normalized by the first heating cycle of PW@NCs (The inset is the DSC curves of PW@NCs with 1, 50, and 100 melting and cooling cycles, respectively).

As shown in Supplementary Fig. 17 and the inset chart, the two melting/crystallization temperature (T_m/T_c) that respectively correspond to the solid/solid and solid/liquid phase change is not affected by the hybridization of rGO or CNT. In contrast, the melting/crystallization enthalpy (ΔH_m/ΔH_c) of PW@NCs slightly decreases as the increase in a

weight ratio of CNT. When the weight ratio of non-working substance (CNT and rGO) increasing to 4.1 wt% (Supplementary Fig. 18), the thermal energy storage density keeps a large value of 152.1 J g^{-1}. The thermal reliability of PW@NCs was investigated by repeating the heating and cooling scans at a rate of 10 °C/min for 100 cycles. The identical phase-change enthalpy demonstrated the excellent reversibility, efficiency, and stability of PW hybridized NCs.

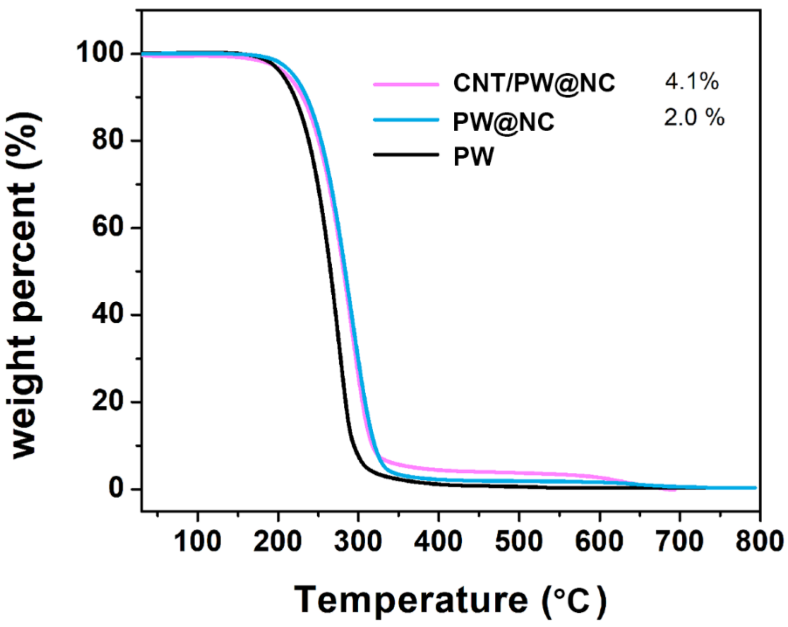

Supplementary Fig. 18 TGA curves of PW, PW@NCs, and CNT/PW@NCs.

Calculation of light-to-heat and energy storage efficiency *η*

The *η* was calculated according to the following **Supplementary Equation 1**[2]

$$\eta = \frac{m \cdot \Delta H}{P \cdot S \cdot (t_e - t_0)}$$

where m is the total mass of the sample, ΔH is the phase change enthalpy obtained by DSC,

P is the light irradiation intensity of simulated light source (100 mW cm^{-2}), S is the surface area of the sample, t_0 and t_e are the light-driven phase change time of the sample before and after the phase change

For woodpile-patterned PW@NCs, under solar irradiation (P = 100 mW cm^{-2}), the phase change time was about 98 s ($t_e - t_0$: the plateau of the gray curve, and labeled by the gray band), the irradiation surface area S (whole cubic scaffold) was about 2.89 cm^2, so the received solar energy was about 28322 mJ (100*2.89*98); the mass (m) of ASF is about 181.8 mg, the phase change enthalpy (ΔH) was 151.9 J g^{-1}, so the storage energy was about 27615 mJ; then the thermal energy conversion and storage efficiency was ～97.5%.

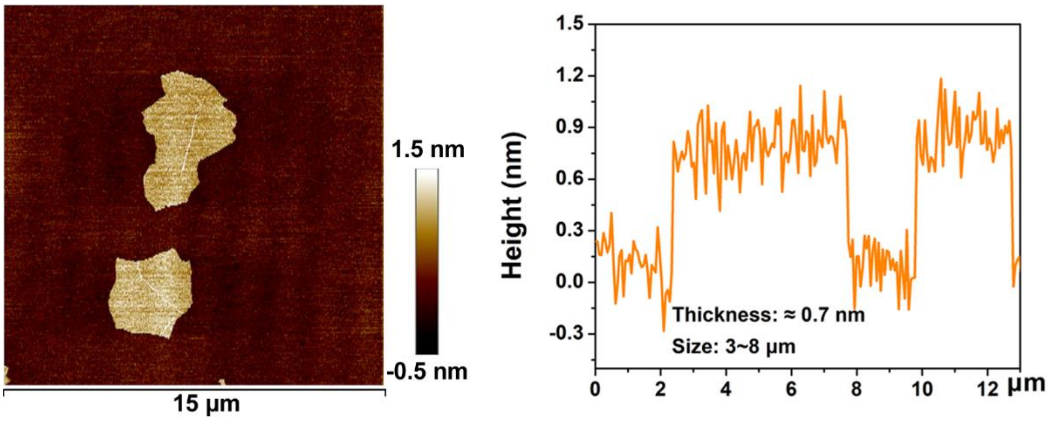

Supplementary Fig. 19 AFM image of GO sheets and height profile

Supplementary videos

Shape changing process of in-plane spiral PW@NCs.

Supplementary References

[1] Y. Li, X. Liu, Z. Zhang, S. Zhao, G. Tian, J. Zheng, D. Wang, S. Shi, T. P. Russell, *Angew. Chem. Int. Ed. Engl.* **57**, 13560 (2018).

[2] G. Li, G. Hong, D. Dong, W. Song, X. Zhang, *Adv. Mater.* **30**, e1801754 (**2018**).
