# Supplementary material for: Interfacial jamming reinforced Pickering emulgel for arbitrary architected nanocomposite with connected nanomaterial matrix
Source: Nat Commun. 2021 Jan 4;12:111. doi: 10.1038/s41467-020-20299-6 (PMC7782697; doi:10.1038/s41467-020-20299-6)
Supplement: Supplementary file 2 — Description of Additional Supplementary Files [file 41467_2020_20299_MOESM2_ESM.pdf]

Description of Additional Supplementary Files

File Name: Supplementary Movie 1

Description: Supplementary Movie 1 displays the shape-changing process of in-plane spiral PW@NCs, which includes 2D to 3D shape-change at 80 °C under gravity, shape-fixation at room temperature and re-heating induced shape reversion.
